# Breast cancer survivors’ opinion on personalizing endocrine therapy and developing informative tools

**DOI:** 10.1038/s41523-024-00655-1

**Published:** 2024-06-17

**Authors:** Elie Rassy, Chiara Benvenuti, Sarra Akla, Antonio Di Meglio, Elise Martin, Julie Havas, André Rieutord, David Combarel, Léonor Fasse, Florian Scotté, Laure Guéroult Accolas, Guillemette Jacob, Anne Bergougnoux, Suzette Delaloge, Ines Vaz-Luis, Barbara Pistilli

**Affiliations:** 1 Medical Oncology Department, Gustave Roussy, Villejuif, France; 2 Medical Oncology Department, Humanitas Research Hospital, Rozzano, Italy; 3 Survivorship Program, INSERM Unit U981, Gustave Roussy, Villejuif, France; 4 Pharmacy Department, Gustave Roussy, Villejuif, France; 5 Department of Pharmacology, Gustave Roussy, Villejuif, France; 6 Interdisciplinary Patient Pathway Division, Gustave Roussy, Villejuif, France; 7 Mon réseau cancer du sein, Paris, France; 8 Les Seintinelles, Paris, France; 9 Odyssea Foundation, Paris, France

**Keywords:** Breast cancer, Pain

## Abstract

Understanding breast cancer survivors’ perspectives is critical to personalizing endocrine therapy (ET) in the adjuvant setting. A nationwide survey among breast cancer survivors was proposed in France, in collaboration with patient advocacy organizations, to assess their perspectives on personalizing ET and developing dedicated informative tools. This survey explored patients’ preferences regarding ET intake schedule, formulation, presentation (color, taste, shape, size, design, and packaging), combination with agents targeting ET-related adverse events, and a mobile application to support them during ET. Of the 1103 individuals who started the survey, 939 (85.1%) were eligible for enrollment and completed the survey. The majority of the participants considered that a personalized ET should take into consideration the intake schedule (*n* = 974, 90.7%) and swallowable tablet formulation (*n* = 606, 64.5%), without a preference for ET presentation (*n* = 619; 65.9%). The majority of the participants expressed a willingness to participate in a potential clinical trial evaluating the combination of ET with agents targeting ET-related adverse events at the start of ET (*n* = 752, 80.1%) or in the case of major ET-related adverse events (*n* = 778, 82.8%). The primary considerations were to have an uncompromised ET efficacy and a guaranteed reduction of adverse events. Last, a dedicated mobile application was considered helpful by 665 participants (70.8%). Informative tools should focus on the recommendations for dealing with adverse events (*n* = 593, 63.2%), the impact on the patient’s daily life (*n* = 515, 54.9%), benefits (*n* = 504, 53.7%), and adverse events (*n* = 494, 52.6%) of ET. This survey paves the way for multimodal strategies that can include a personalized ET (e.g., ET in combination with agents targeting ET-related adverse events) and dedicated mobile applications to ultimately improve adherence.

## Introduction

Breast cancer is the most frequently diagnosed cancer among women and ranks as the second leading cause of cancer-related deaths^1^. The widespread implementation of screening strategies has resulted in a higher proportion of early-stage breast cancers that are managed with a curative intent. Seventy percent of individuals diagnosed with breast cancer have tumors that express hormone receptors (HR + BC), necessitating endocrine therapy (ET), a cornerstone of the treatment strategy^2^. The meta-analysis performed by the Early Breast Cancer Trialists’ Collaborative Group (EBCTCG) evaluating individual patient-level data from multiple clinical trials have shown that adjuvant ET almost halved the recurrence rates and reduced by approximately one-third the mortality rates^3^. However, adjuvant ET, prescribed for 5 years or more, is associated with adverse events that impact the patient’s quality of life, subsequently reducing adherence to treatment^4,5^. Non-adherence and early discontinuation of ET occur in 30–50% of breast cancer survivors^6–8^. We have previously reported that 16.0% of premenopausal patients with HR + BC were non-adherent to tamoxifen after one year of treatment^9^. Biochemically proven non-adherent patients had a higher risk of distant recurrences (HR 2.31, 95% CI 1.05–5.06) shortly after treatment discontinuation or interruption^9^.

Non-adherence is commonly multifactorial and primarily affects patients at the extremes of age^6,10^, those with low socioeconomic status^11–13^, patients with limited knowledge about the benefit of adjuvant ET^7,14^, and intolerable adverse events, mainly menopausal symptoms^15–17^. In a recently reported meta-analysis, lowering medication costs and a subgroup of psychosocial and reminder interventions had a significant impact on promoting adherence^18^. Nevertheless, it remains necessary to understand the levers that could facilitate adherence to ET from the patient’s perspective. Therefore, we conducted a survey among HR + BC survivors who were either receiving or had previously received adjuvant ET to explore their views on personalizing ET (schedule, formulation, presentation, and combination with agents targeting ET-related adverse events) and developing dedicated informative tools.

## Results

### Patient characteristics

Among the 1103 individuals who started the survey, 1051 (95.3%) were eligible for enrollment, of whom 939 (85.1%) completed the survey. Participants were predominantly females (*n* = 933; 99.4%), with the majority (*n* = 593; 63.2%) aged 50 years and above and a minority (*n* = 7; 0.8%) younger than 30 years (Table 1). At the time of survey completion, 629 participants were receiving adjuvant ET and 222 had completed their treatment (67.0% and 23.6% respectively). Seventy-three participants had discontinued their adjuvant ET prematurely due to adverse events that they found unacceptable and 15 participants stopped for personal reasons (7.8% and 1.6% respectively). The duration of ET already received at the time of the survey was predominantly between 3 and 5 years followed by 1 and 3 years for 362 (38.6%) and 281 participants (29.9%), respectively. A smaller proportion of participants had an ET duration shorter than 1 year (*n* = 150, 16.0%) and exceeding 5 years (*n* = 146, 15.6%).

**Table 1:** Breast cancer survivors’ characteristics (*n* = 939)

Variables	Number of participants (%)
*Gender*	
Male	6 (0.9%)
Female	933 (99.1%)
*Age category at the time of survey completion*	
<30 years	7 (0.8%)
30–39 years	84 (9.0%)
40–49 years	255 (27.2%)
50–59 years	309 (32.9%)
60–69 years	227 (24.2%)
>70 years	57(6.1%)
*Status of endocrine therapy*	
Ongoing treatment	629 (67.0%)
Completed treatment	222 (23.6%)
Discontinued for poor tolerance	73 (7.8%)
Discontinued for personal reasons	15 (1.6%)
*Duration of endocrine therapy*	
Less than 6 months	63 (6.7%)
Between 6 months and 1 year	87 (9.3%)
Between 1 and 3 years	281 (29.9%)
Between 3 and 5 years	362 (38.6%)
More than 5 years	146 (15.6%)

### Survey results

**Fig. 1:**
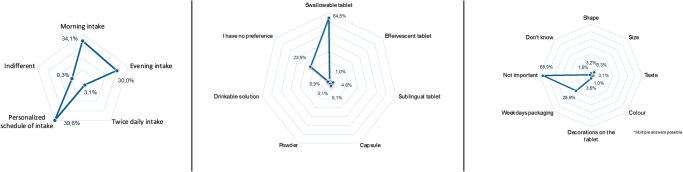
The survey’s key findings on endocrine therapy preferences. Preferences for endocrine therapy intake schedule (left), formulation (middle), and presentation (right).

Participants showed no clear preference for the ET intake schedule, with approximately equal numbers favoring morning and evening hours (*n* = 320 [34.1%] and 282 [30.0%]), 29 participants (*n* = 29, 3.1%) preferred twice-daily intake and 372 (39.6%) preferred to know the most suitable time for them to take the ET. 606 respondents favored a swallowable tablet (*n* = 606, 64.5%) over a capsule (*n* = 48, 5.1%) or a sublingual tablet (*n* = 43, 4.6%), while a quarter of participants felt this aspect was not as relevant to them (*n* = 224, 23.9%). About two-thirds of the participants (*n* = 619, 65.9%) believed that the color, taste, shape, size, design, and packaging were not relevant. Two-hundred and seventy-one participants (28.9%) showed a preference for blister packaging relevant to weekdays, whereas other preferences were less common (Fig. 1).

**Fig. 2:**
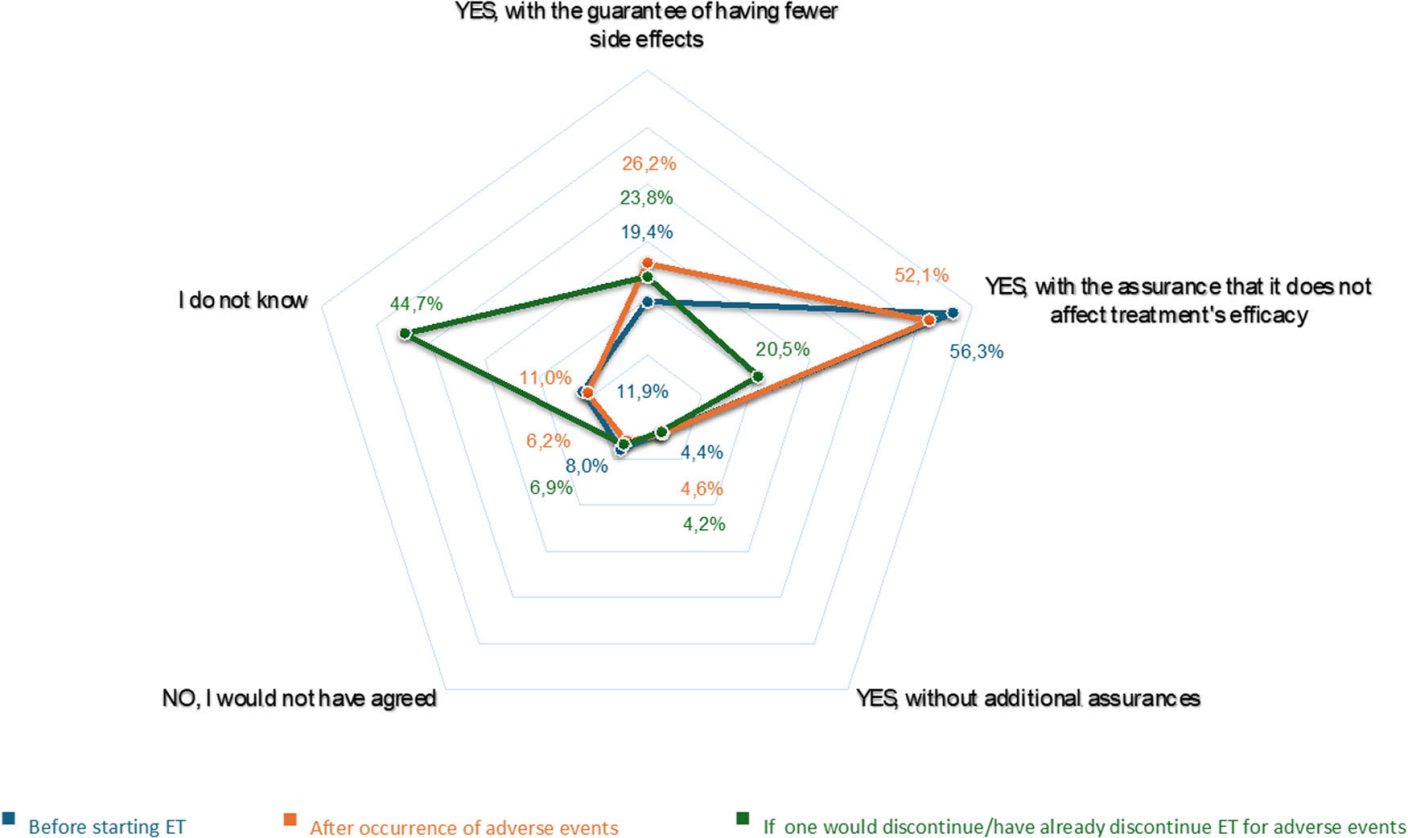
Willingness to participate in a potential clinical trial evaluating a drug that would combine endocrine therapy and an active ingredient directed against endocrine therapy-related adverse events.

The majority of the participants (*n* = 703, 75.2%) were interested in having a potential drug that would combine ET with an active ingredient directed against ET-related adverse events. Concerning the participation in a clinical trial for such a drug, 752 respondents (80.1%) would have enrolled at the time of starting ET, and 778 respondents (82.8%) expressed their willingness to participate in such trial in the case of major ET-related adverse events. Among participants willing to enroll in such clinical trials at the time of starting ET, the primary considerations were not to compromise ET efficacy and a guaranteed reduction of adverse events (*n* = 529 [56.3%] and 182 [19.4%]). Similarly, patients willing to participate in such trials in the case of major ET-related adverse events privileged the same considerations (*n* = 489 [52.1%] and 182 [19.4%] and 246 [26.2%], respectively) (Fig. 2).

**Fig. 3:**
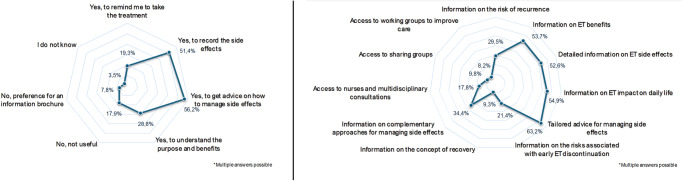
The survey’s key findings on dedicated mobile applications and informative tools. Content and use purposes of a dedicated mobile application (left) and informative tools (right).

The majority of participants (*n* = 665, 70.8%) expressed interest in a potential dedicated mobile application for ET purposes, while 17.9% (*n* = 168) did not consider this useful, and 7.8% (*n* = 73) favored a brochure. The main reasons for this interest were the desire for detailed information (*n* = 528, 56.2%) and to record adverse events to discuss them with the physician (*n* = 483, 51.4%). Participants considered that informative tools should focus on the recommendations for dealing with adverse events (*n* = 593, 63.2%), the impact on the patient’s quality of life (*n* = 515, 54.9%), benefits (*n* = 504, 53.7%) and adverse events (*n* = 494, 52.6%) of ET. Less common motives included obtaining comprehensive information on complementary medicine techniques to manage adverse events (*n* = 323, 34.4%), risks of disease relapse, and early treatment discontinuation (*n* = 277 [29.5%] and 201 [21.4%], respectively) (Fig. 3).

## Discussion

Adjuvant ET has reduced the risk of relapse for patients with HR + BC but is commonly associated with troublesome treatment-related adverse events, especially musculoskeletal and menopausal symptoms, that may compromise compliance to treatment and, subsequently, its efficacy^4,19^. Indeed, non-adherence to ET is frequently observed and significantly increases from 6% to 42% between the 1st and 5th year of treatment, with a negative impact on survival outcomes^20^. Active efforts to improve treatment adherence have yielded varying results, with several interventions, such as motivational statements about the effectiveness of ET and recommendations to encourage adherence failing to improve adherence^21–24^. A recent meta-analysis focusing on interventions aimed at improving adherence to ET found that several strategies, particularly behavioral targeting modifiable risk factors, effectively enhanced compliance to treatment^18^. In this context, the perspective of patients who have experienced ET first-hand could be a valuable input to guide the development of personalized ET and dedicated mobile applications that can be tailored to overcome the barriers to optimal adherence exactly where patients have encountered the greatest challenges. Therefore, to better explore and leverage the perspective of HR + BC patient survivors, we developed a comprehensive survey in collaboration with national breast cancer patient advocacy organizations in France to explore the patient’s perspectives on developing personalized ET and dedicated informative tools.

Participants expressed a clear preference for a personalized ET in terms of intake schedule and ET formulation, with a lower preference for ET color, taste, shape, size, design, and packaging. They also showed a proactive attitude towards experimental efforts aimed at improving the tolerability of ET overall and in the event of major ET-related adverse events. Notably, the main considerations were to primarily ensure an uncompromised efficacy for the ET and, secondly, a guaranteed reduction of adverse events. These considerations highlight the important of participatory research that ensures capacity building and training of research participants to fully understand the research focus and for investigators to design studies that respond to patient needs^25–27^. Our findings corroborate that the management of adverse events is a key cornerstone in overcoming the lack of adherence to treatment, as they are often cited as the main reason for discontinuation and interruption of treatment. Participants considered that informative tools should focus on the recommendations for dealing with adverse events (n = 593, 63.2%), the impact on the patient’s daily life (*n* = 515, 54.9%), benefits (*n* = 504, 53.7%) and adverse events (*n* = 494, 52.6%) of ET.

Last, 665 participants (70.8%) considered a dedicated mobile application to be a helpful vehicle for information and eventually self-management. Digital tools, such as dedicated mobile applications tailored to the individual needs of patients, could alleviate the potential sense of neglect that patients feel in the case of less frequent in-person follow-ups, without placing an additional burden on the healthcare system^28^. A preference for having a dedicated mobile application that could assist in managing adverse events and provide information about the benefits and adverse events of ET strongly emerged from our survey and aligns with findings from other studies. On one hand, patients want to take an active role in their treatment and seek comprehensive awareness of all aspects of ET to better navigate the complexities of their treatment journey. Being well-informed about the side effects and benefits of treatment fosters a sense of control and helps patients to better tolerate, manage, and cope with their ET^29–31^. Nevertheless, this was not consensual, with some participants (*n* = 168, 17.9%) finding the application useless. In a large cohort study, the use of a dedicated application covering the decision of the multidisciplinary team discussion and generated the treatment process, reminding patients of their treatment, and confirming treatment completion was not significantly associated with adherence (*p* = 0.54)^32^. Our survey showed that participants valued other purposes for the application, which highlights the importance of personalizing the application according to the patient’s perspective. A particular observation also stemmed from our survey as almost half the respondents (*n* = 483, 51.4%) considered the application valuable for documenting adverse events in order to discuss them with the oncologist. This highlights the application’s potential role as a supplement, rather than a replacement for, the patient-oncologist relationship while serving as a tool to enhance the quality of communication. This perspective could explain the findings of the THRIVE study (NCT03592771), reporting that a dedicated application combined with tailored messages led to better self-reported mental health and fewer high-cost encounters although it did not improve adherence to ET, symptom burden, self-efficacy in managing symptoms, or office visits^33^.

Overall, this survey contributes to our understanding of the complex and multifaceted issue of non-adherence and paves the way for future research in this area. Building on the findings of this survey and previous data from the CANTO cohort, we hypothesized that a multimodal approach combining a personalized drug combining ET with a pharmacological agent targeting ET-related adverse events and multi-component, multi-level mobile application that provides personalized supportive and educational information for ET adherence could be of interest. Such a study would fit within several other ongoing trials that use multimodal strategies including digital therapeutics to address the intricate granularity and multiple facets underlying endocrine treatment non-adherence in early breast cancer patients such as COMPLIANCE (NCT04176809), WEBAPPAC (NCT04554927), REACH (NCT03980093) trials and others (including NCT04054557 and NCT04379570).

This survey has several notable strengths. It was designed in collaboration with patient advocacy organizations, ensuring that the perspectives and needs of breast cancer survivors were effectively addressed. It targeted a large population of subjects and explored the breast cancer survivor’s perspectives in regard to the personalization of the adjuvant ET as well as the information required to enhance adherence, in order to allow a generalizability of the reported findings. It is also important to acknowledge several limitations of this survey. The number of participants who were sent the survey was not available; thus, the response rate cannot be estimated. Important data regarding sociodemographic factors, comorbidities, and the specific type of ET (tamoxifen vs aromatase inhibitors with or without GnRH analogs), known to impact adherence to ET, were not collected^34^. Furthermore, the survey did not gather information regarding the time of diagnosis and the time elapsed since the completion of ET among the participants who had already completed ET, which may contribute to a selection bias. Additionally, the low representation of young and old patients (patients < 30 years *n* = 7 [0.8%] and those >70 years *n* = 57 [6.1%]) limits the generalizability of our findings to these demographic groups and points out the need for tailored strategies to address them. Participants who discontinued treatment early for tolerance/personal reasons could be less likely inclined to answer the survey, explaining the low rate of nonadherence and early discontinuation of ET in our cohort (*n* = 88; 9.4%) in comparison to the published literature^9,35^. The survey did not assess subjective (patient-reported) or biologic (hormonal levels) measures of adherence to the prescribed therapy, nor did it evaluate compliance with the prolonged use of the application. Moreover, the majority of the participants were insured by the French social security system, which may limit the generalizability of the findings to uninsured populations or those within different healthcare systems. Depending on the healthcare system and personal insurance policy, the costs for developing and implementing such a mobile application may prevent its widespread availability to patients. Last, the recruitment of participants via email and among patient associations, which typically include proactive individuals more prone and accustomed to innovations and digital tools, inherently influenced how these individuals perceive the use of the applications.

In conclusion, this survey has provided valuable insights into the perspectives of breast cancer survivors and unraveled potential grounds that could improve adherence. Participants expressed a preference for personalized ET, particularly in terms of intake schedule and formulation, while placing less emphasis on the shape, size, color, or taste design and packaging. Furthermore, the participants expressed interest in the development of a single drug that combines ET with an active ingredient designed to address potential adverse events without compromising the ET efficacy. Last, participants consider that informative tools, mainly mobile applications, would be highly helpful in addressing their inquiries regarding the benefits and adverse events of ET. Several ongoing multimodal efforts hold promise in potentially improving adherence and, subsequently, patients’ outcomes.

## Methods

### Study design

This observational cross-sectional survey was conducted in collaboration with three major French breast cancer patient advocacy organizations “Les Seintinelles”, “Mon Réseau Cancer Du Sein” and “Odyssea”. Sentinelles is a nonprofit, community-driven research platform developed in collaboration with psycho-oncologists that aims to empower patients and involve them actively in cancer research efforts^36^. Mon Réseau Cancer Du Sein is a secure social platform born from the “Patients en réseau” association and designed to encourage patients to share their experiences and to support exchanges between patients or between relatives^37^. Odysséa is an association whose mission is to mobilize women, men, and children by organizing sports events to raise funds for the fight against breast cancer^38^.

After an extensive literature review on the concerns of HR + BC patients regarding adjuvant ET, a comprehensive survey was designed to explore the participants’ views on what could improve their adherence to adjuvant ET and their opinions on how to mitigate adverse events associated with this form of therapy. The first draft was discussed with the three breast cancer patient advocacy organizations—“Seintinelles”, “Mon Réseau Cancer Du Sein” and “Odyssea” and a consensual draft was approved (Supplementary material).

The final version of the survey contains four sections. The first section (items 1–5) addressed the participants’ history of breast cancer treated with ET, gender, age category at the time of the survey completion, status concerning ET (ongoing treatment, completed treatment, stopped for poor tolerance, and discontinuation for personal reasons), and duration of prescribed ET. The second section (items 6–8) included three items concerning patient preferences concerning the ET intake schedule, presentation (color, taste, shape, size, design, and packaging), and formulation (pill, effervescent, sublingual, tablet, powder, and liquid). The third section (items 9–12) explored the interest of participants in a potential drug that would combine ET and an active ingredient directed against ET-related adverse events. The last section (items 13 and 14) included two items reflecting on the input and content of a potential mobile application that would have been or would be helpfully used to support patients throughout ET. The final version of the survey was approved by the investigators and addressed to breast cancer survivors in the Seintinelles network by email.

The study enrolled participants throughout France between July 1st and July 31st, 2020. Eligible subjects were patients with a previous diagnosis of HR + BC who received at least one month of ET at any point during their treatment, either ongoing or have completed ET. Individuals within the “Seintinelles” network were sent a link via email to anonymously complete a one-time survey via the SurveyMonkey interface. Each participant was asked to fill out the survey only once, and this was tracked based on their Internet Protocol address. No reminders or second invitations were sent to any of the participants.

An information letter was provided to the participants, clearly stating that by completing the survey, they were implicitly agreeing to participate in the study, and subsequently, written consent was not required. The study was examined and approved by the internal review board at Gustave Roussy, Villejuif, France on July 3rd, 2020 as well as the data protection department. All research procedures and practices conducted as part of this survey were carried out in accordance with established ethical standards and regulations.

### Statistical analysis

Given that the main focus of the survey was to collect the input of breast cancer survivors concerning the development of personalized ET and dedicated informative tools, descriptive statistics using frequency and proportion of qualitative variables were used to describe the participants’ socio-demographic and clinical characteristics.

### Supplementary information

Survey (Supplement)

Related Manuscript File

## Data Availability

The survey data collected and/or analyzed as part of the current study are not publicly available but may be obtained from the corresponding author upon reasonable request.
