# Supplementary material for: Breast cancer survivors’ opinion on personalizing endocrine therapy and developing informative tools
Source: NPJ Breast Cancer. 2024 Jun 17;10:48. doi: 10.1038/s41523-024-00655-1 (PMC11183231; doi:10.1038/s41523-024-00655-1)
Supplement: Supplementary file 1 — Survey (Supplement) [file 41523_2024_655_MOESM1_ESM.pdf]

Section 1	Item 1	Vous avez eu un cancer du sein et vous avez suivi pendant au moins un mois un traitement par hormonothérapie.	Oui
			Non
	Item 2	Vous êtes	Un homme
			Une femme
	Item 3	Quel âge avez-vous ?	< 30
			30-39
			40-49
			50-59
			60-69
			>70
	Item 4	Actuellement :	Vous êtes en cours de traitement par hormonothérapie
			Vous avez terminé votre traitement d'hormonothérapie
			Vous avez arrêté l'hormonothérapie car les effets secondaires étaient inacceptables pour vous
			Vous avez arrêté l'hormonothérapie par choix personnel
	Item 5	Depuis quand prenez-vous ce traitement ou pour combien de temps avez-vous pris ce traitement ?	Moins de 6 mois
			Entre 6 et 12 mois
			Entre 1 et 3 ans
			Entre 3 et 5 ans
			Plus de 5 ans
Section 2	Item 6	Quel est votre avis sur la prise de l'hormonothérapie que vous recevez ou avez reçue (plusieurs réponses possibles) :	Je préfère/préférais prendre mon hormonothérapie en 1 fois le matin
			Je préfère /préférais prendre mon hormonothérapie en 1 fois le soir
			Je préfère/préférais prendre mon hormonothérapie en 2 fois matin et soir
			J'aimerai/j'aurais aimé savoir quel est le moment le plus adapté pour moi pour prendre l'hormonothérapie

			Je n’ai pas d’avis
	Item 7	Si c’était possible de choisir le type de prise, je préférerai/ j’aurais préféré :	Un comprimé à avaler
			Un comprimé effervescent ou dispersible dans l’eau
			Un comprimé sublingual (à dissoudre sous la langue)
			Une gélule
			Un sachet (poudre à dissoudre)
			Une solution buvable
			Je n’ai pas de préférence
	Item 8	Quel est votre avis sur la présentation des comprimés d’hormonothérapie (forme et couleur, goût) :	La forme des comprimés (rond, ovale, …)
			La taille des comprimés (plus petit que l’existant quitte à devoir en prendre plusieurs)
			Le goût des comprimés
			La couleur des comprimés
			D’avoir un mini visuel au choix imprimé sur les comprimés (trèfle à 4 feuilles, rose, sourire, …)
			Un type d’emballage (blister avec jours de la semaine indiqués)
			Je ne pense pas que ce soit important
			Je ne sais pas
Section 3	Item 9	Si vous avez/aviez des effets secondaires, aimeriez-vous/auriez-vous aimé prendre un comprimé d'hormonothérapie qui contient aussi un médicament pour traiter ces effets secondaires ?	Oui
			Non
	Item 10	Auriez-vous été d’accord pour participer à un tel essai clinique au tout début de votre traitement d’hormonothérapie ?	Oui avec la garantie d’avoir moins d’effets secondaires
			Oui avec la garantie que cela n’affecte pas l’efficacité du traitement
			Oui sans garantie sur l’efficacité du traitement ou sur les effets secondaires
			Non je n’aurais pas accepté
			Je ne sais pas
	Item 11		Oui avec la garantie d’avoir moins d’effets secondaires

		Seriez-vous/ Auriez-vous été d'accord pour participer à un tel essai clinique après que vous ayez expérimenté des effets secondaires importants ?	Oui avec la garantie que cela n'affecte pas l'efficacité du traitement
			Oui sans garantie sur l'efficacité du traitement ou sur les effets secondaires
			Non je n'aurais pas accepté
			Je ne sais pas
	Item 12	Si vous avez arrêté l'hormonothérapie avant la fin de votre prescription, auriez-vous été d'accord pour participer à un tel essai clinique si on vous l'avait proposé au moment où vous alliez arrêter	Oui avec la garantie d'avoir moins d'effets secondaires
			Oui avec la garantie que cela n'affecte pas l'efficacité du traitement
			Oui sans garantie sur l'efficacité du traitement ou sur les effets secondaires
			Non je n'aurais pas accepté
			Je ne sais pas
Section 4	Item 13	Pensez-vous qu'une application mobile vous serait/aurait été utile pour vous accompagner tout au long de votre traitement d'hormonothérapie ? (plusieurs réponses possibles)	Oui, pour me rappeler l'heure de prise du traitement
			Oui, pour enregistrer les effets secondaires de l'hormonothérapie et pouvoir en discuter avec mon médecin
			Oui, pour retrouver des conseils pour gérer les effets secondaires de l'hormonothérapie
			Oui, pour comprendre l'intérêt de ce traitement et les bénéfices attendus
			Non, ne m'aurait été/ serait d'aucune utilité
			Non, j'aurais préféré/je préférerais une brochure d'information
			Je ne sais pas
	Item 14	A votre avis quels sont les 4 contenus les plus importants qui devraient être présents dans une «application mobile » ou dans une brochure accompagnant l'hormonothérapie	Des informations sur les risques de récidive et les facteurs impliqués (envahissement ganglionnaire, le grade, etc…)
			Des informations claires sur les bénéfices de l'hormonothérapie
			Des informations détaillées sur les effets secondaires de l'hormonothérapie

			Des informations sur l'impact de l'hormonothérapie sur la vie quotidienne (estime de soi, acceptation de soi, humeur, énergie, concentration, sexualité, …)
			Des conseils adaptés pour la gestion des effets secondaires
			Des informations sur les risques en cas d'interruption précoce de l'hormonothérapie
			Des informations sur les approches complémentaires pour la gestion des effets secondaires
			Des informations sur le concept de rétablissement « il ne suffit pas d'être déclaré(e) guéri(e) pour être rétabli(e) »
			Des accès à des infirmières et à des consultations multidisciplinaires
			Des accès à des groupes de partage
			Des accès à des groupes de travail pour améliorer la prise en charge
